# Research note: Increased lipid accumulation within broiler preadipocytes during differentiation in vitro at atmospheric oxygen tension

**DOI:** 10.1016/j.psj.2024.104531

**Published:** 2024-11-08

**Authors:** Jéssica Moraes Cruvinel, Elizabeth S. Greene, Rachel W. Read, Kichoon Lee, Sami Dridi, Paula R. Chen

**Affiliations:** a Division of Animal Sciences, University of Missouri, Columbia 65211, USA; b Department of Animal Sciences, The Ohio State University, Columbus, OH 43210, USA; c University of Arkansas, Center of Excellence for Poultry Science, Fayetteville, AR 72701, USA; d USDA-ARS, Plant Genetics Research Unit, Columbia, MO 65211, USA

**Keywords:** Adipogenesis, Lipids, Broiler, Oxygen tension, Preadipocyte

## Abstract

In the broiler industry, intensive genetic selection has been placed on muscle growth which has undesirably led to increased fat accretion. Models of chicken preadipocyte differentiation *in vitro* have conventionally used incubators without the ability to control oxygen (O_2_) tension; thus, the cells are exposed to atmospheric (∼20-21%) O_2_, which is supraphysiological compared to the O_2_ tension within adipose tissue. The objective of this study was to investigate embryonic broiler preadipocyte differentiation at different O_2_ tensions, including atmospheric (20%), physiological (5%), and hypoxic (1%). Culture at 1% O_2_ resulted in increased abundance of HIF1α, a canonical protein stabilized during hypoxia, thus confirming effectiveness of the treatment. Increased accumulation of lipid was observed in preadipocytes cultured in adipogenic differentiation medium compared to the control medium. When considering oxygen tension, lipid accumulation was increased in preadipocytes that were cultured in differentiation medium at 20% O_2_ compared to 5% or 1% O_2_. Furthermore, abundance of transcripts related to fatty acid transport and adipogenesis, fatty acid binding protein 4 (*FABP4*) and peroxisome proliferator-activated receptor gamma (*PPARγ*), were increased in differentiated preadipocytes cultured at 20% O_2_ compared to 5% or 1% O_2_. Abundance of transcripts related to lipid synthesis and oxidation, acyl-CoA synthetase long chain family member 1 (*ACSL1*) and carnitine palmitoyltransferase 1A (*CPT1A*), were increased in the differentiation cultures compared to the control cultures. Abundance of glutathione peroxidase 4 (*GPX4*) was increased in all the differentiation cultures compared to the controls, regardless of oxygen tension; however, differences in the abundance of other antioxidant enzymes were not observed. Overall, exposure to atmospheric oxygen tension promotes lipid accumulation within chicken preadipocytes, which may need to be considered when developing *in vitro* models of this process.

## Introduction

Genetic selection of broiler chickens has resulted in animals that outperform all other breeds regarding feed conversion and skeletal muscle deposition; however, this high metabolic rate has also triggered various metabolic syndromes, such as ascites, bone disorders, muscle myopathies, insulin resistance, and obesity (Wang et al., 2017; Emami et al., 2021). The progression of obesity in broilers is directly linked to their unregulated feed intake and hyperphagy, and excessive adipose tissue accumulation decreases overall feed efficiency, carcass yield, and product quality, and consequently, impacts profitability and acceptability by consumers.

Oxygenation of tissues is an important factor to keep cellular metabolism functioning appropriately. Albeit in humans, the oxygen tension of healthy adipose tissue ranges from 3-11%, and tensions below this range can indicate hypoxia (Lempesis et al., 2020). Rapid expansion of adipose tissue has been studied in models of severe obesity, and dysregulation of oxygenation, either hypoxia or hyperoxia, has been observed to be involved in the progression of this disease (Goossens et al., 2011; Trayhurn, 2013). On one hand, excessive hyperplasia and hypertrophy of adipocytes in mouse models of obesity has been shown to impair adipose vascularization, which restricts the supply of oxygen (Trayhurn, 2013). This state of hypoxia elicits a myriad of transcriptional factors, such as hypoxia-inducible factor 1 alpha (HIF1α), that can prompt an inflammatory response and lead to metabolic syndromes, including insulin resistance. Contrarily, Goossens et al. (2011) noted a hyperoxic state in adipose tissue of humans who were obese, which was determined to be the result of diminished vascularization and blood flow as well as decreased oxygen consumption by the adipocytes. Hyperoxia can increase the intracellular levels of reactive oxygen species (ROS), which may lead to membrane and DNA damage, further exacerbating tissue dysfunction. To date, there have been no studies regarding the role of oxygen in adipocyte differentiation and the progression of obesity in broilers.

Importantly, the generation of *in vitro* models to study adipogenesis in chickens has been critical to provide insight into the molecular and cellular changes during this process. Although the medium conditions for adipogenic differentiation of chicken preadipocytes have been optimized (Kim et al., 2021), other external factors of the artificial environment, such exposure to differing oxygen tensions, have not been investigated. In addition, as conflicting results have been reported regarding the role of oxygen tension during adipogenesis and lipogenesis (Goossens et al., 2011; Trayhurn, 2013), the objective of this study was to investigate the differentiation trajectory of broiler preadipocytes at different oxygen tensions.

## Materials and methods

### Preadipocyte isolation

Use of chicken embryos were in accordance with approved protocol and standard operating procedures by the Animal Care and Use Committee of the University of Missouri (Protocol 38126). Fertile Cobb 500 broiler eggs were obtained from Cobb-Vantress (Siloam Springs, AR), incubated until day 18, and euthanized by CO_2_ exposure for 20 min followed by decapitation. Preadipocytes were isolated at embryonic day 18 by excising abdominal adipose tissue, mincing, digesting in 0.5 mg/mL of collagenase type IV (Sigma Aldrich, St. Louis, MO) for 3 h at 38°C, and filtering through a 70-μm cell strainer. The stromal-vascular fraction containing preadipocytes was separated from differentiated adipocytes and pelleted by centrifuging at 300 × g for 5 min. Preadipocytes were expanded in normal growth media containing Dulbecco's modified Eagle's medium and Ham's F-12 nutrient mixture (DMEM/F12, Thermo Fisher, Waltham, MA) with 10% fetal bovine serum (FBS, Sigma-Aldrich) on collagen-coated plates.

### Adipogenic differentiation and culture

Control cultures were maintained in normal growth medium, which consisted of DMEM/F12 with 10% FBS. For differentiation cultures, adipogenic differentiation was induced by replacing normal growth medium with DMEM/F12 containing 10% chicken serum (CS, Thermo Fisher), 10 μg/mL linoleic acid (Sigma-Aldrich), 20 μg/mL oleic acid (Sigma-Aldrich), and 1 μg/mL insulin (Sigma-Aldrich) at approximately 70% confluence (Kim et al., 2021). Control and differentiation cultures were placed in humidified incubators at either 20-21% O_2_ (atmospheric, herein referred to as 20%) or 5% O_2_ (physiological) with 5% CO_2_ at 38°C for 3 days. Rapidly switching culture conditions from 20% O_2_ to 1% O_2_ can lead to disruption of homeostasis and widespread necrosis of the cells, known as hypoxic shock. To prevent hypoxic shock in the control and differentiation cultures at 1% O_2_, a step-down approach was used where cultures were placed at 5% O_2_ at the beginning of day 1, 2.5% O_2_ at the beginning of day 2, and 1% O_2_ at the beginning of day 3. The duration of hypoxia exposure was based on previous studies (Lempesis et al., 2020; Emami et al., 2021). Cells were either fixed in 4% paraformaldehyde for 15 min at room temperature or pelleted and snap frozen in liquid nitrogen followed by storage at -80°C.

### Nile red staining and quantification

Nile red selectively stains neutral lipids within cells and fluoresces yellow or red, allowing for quantification (Greenspan et al., 1985). To visualize lipid accumulation in broiler preadipocytes, fixed cells were washed two times with phosphate-buffered saline (PBS) and stained with the Nile Red Staining Kit according to the manufacturer's instructions (Abcam, Cambridge, MA). Afterwards, cells were washed with PBS, and the nuclei were stained with 10 μg/mL 4′,6-diamidino-2-phenylindole (DAPI) for 10 min. Images were captured by using a Nikon Eclipse Ts2 and NIS elements 5.41.02 software (Nikon Inc., Tokyo, Japan) with a TRITC filter for Nile Red (lipid droplets) and a UV filter for DAPI (nuclei). Relative fluorescence intensity was measured by using Fiji (available at: https://imagej.net/software/fiji/) in a minimum of five images per group, and the Nile Red fluorescence value from each culture was divided by the total cell number to estimate the relative intensity per culture.

### Western blot

Total proteins from the cultures were extracted by using RIPA buffer (Thermo Fisher) with Halt™ protease and phosphatase inhibitor cocktail (Thermo Fisher) and quantified with a Qubit 4 fluorometer (Thermo Fisher). Protein lysates (25 μg per sample) were separated on a 4–20% SDS-PAGE gel and dry transferred to PVDF membranes by using the Trans-Blot Turbo Transfer System (Bio-Rad Laboratories, Hercules, CA). Membranes were blocked with 5% non-fat dry milk in tris-buffered saline with 0.1% Tween 20 (TBST) and incubated with primary antibodies overnight at 4°C. Primary antibodies included rabbit anti-HIF1α (1:1000; LS-C287203; LSBio, Seattle, WA), rabbit anti-hydroxy-HIF1α (1:1000; 3434; Cell Signaling Technology, Danvers, MA), and rabbit anti-glyceraldehyde 3 phosphate dehydrogenase (GAPDH) (1:1000; NB300-327; Novus Biologicals, Centennial, CO). Membranes were washed with TBST and incubated with goat anti-rabbit IgG (1:5000; 31466; Thermo Fisher) for 1 h at room temperature. The signal was visualized by chemiluminescence with the Pierce ECL 2 Western Blotting Substrate (Thermo Fisher) and captured on a ChemiDoc (Bio-Rad Laboratories) with the same exposure times for each replicate. Densitometries were evaluated against GAPDH for normalization by using Fiji.

### RNA extraction, cDNA synthesis, and quantitative real-time PCR

Total RNA from the frozen preadipocyte culture pellets was extracted by using the RNeasy Mini Kit according to the manufacturer's instructions (Qiagen, Germantown, MD). Synthesis of cDNA was performed by using the SuperScript IV VILO Master Mix according to the manufacturer's instructions (Thermo Fisher). Primer sequences used in this study were described previously (Kim et al., 2021) except for carnitine palmitoyltransferase 1A (*CPT1A*, NM_001012898.1, F: 5`- CTGAAGAAGAACCCTGAGATG, R: 5`- TGCTGGAGACATGGAAATG, size: 102 bp), catalase (*CAT*, NM_001031215.2, F: 5`-TACGGAGGTAGAACGATGG, R: 5`- GTGTCAGGATACGCAAAGAG, size: 105 bp), glutathione peroxidase 4 (*GPX4*, NM_204220.3, F: 5`- ACCTCCATCTACGACTTCC, R: 5`- CGCGGTCTTTCCTCATTT, size: 117 bp), peroxiredoxin 3 (*PRDX3*, XM_426543.6, F: 5`- CACCTGGCCTGGATAAATAC, R: 5`- CCTTCTAGCAGAACACCATAAT, size: 116 bp), and superoxide dismutase 2 (*SOD2*, NM_204211.2, F: 5`- TAGCAGCCTGTGCAAATC, R: 5`- AGATAATAGGCATGTTCCCATAC, size: 91 bp). Beta-actin (*ACTB*, NM_205518.2 F: 5`- GCCAACAGAGAGAAGATGAC, R: 5`-CACCAGAGTCCATCACAATAC, size: 130 bp) was used as a housekeeping gene. After diluting cDNA samples to 5 ng/μL, reactions were run in triplicate on a QIAquant 96 5plex (Qiagen) with the conditions 95°C for 3 min, and 35 cycles of 95°C for 10 s, 55°C for 10 s, and 72°C for 30 s. Afterwards, a dissociation curve was generated to ensure that a single product was amplified. The abundance of each transcript was calculated relative to beta-actin (*ACTB*), which was not statistically different between groups (*P* > 0.05), and then normalized to the abundance of the 20% control cultures. The comparative threshold cycle method (2^-ΔΔCt^) was used to determine transcript abundance for each sample.

### Statistical analysis

All experiments were repeated at least three times so that replicate variation could be assessed. Analyses of quantitative data were performed by using SAS software, version 9.4 (SAS Institute Inc., Cary, NC). The Shapiro–Wilk test was used for assessing the normality assumption for each experiment, and data were log transformed if deviation from the normality assumption was detected. Data were analyzed by using a two-way ANOVA with O_2_ tension and medium as fixed factors, and culture as the experimental unit. If significant effects were observed after the ANOVA, the means were compared by Tukey's HSD multiple comparison test. Differences were considered significant at *P* < 0.05.

## Results and discussion

Understanding factors that influence adipogenesis is chickens is critical to improve production efficiency and overall yield. Culture medium components have been identified and optimized to induce adipogenesis in chicken preadipocytes *in vitro*; however, other aspects of the culture environment have not been examined. In the current study, the effects of different O_2_ tensions (20%, 5%, or 1%) on broiler preadipocyte differentiation *in vitro* was investigated. Routinely, cell culture incubators maintain an environment of 5% CO_2_ in air, meaning atmospheric (∼20%) O_2_ tension is present. However, 20% O_2_ does not represent oxygen tensions *in vivo*; thus, 5% O_2_ was selected to represent the physiological tension found within the body (Lempesis et al., 2020). A hypoxic condition of 1% O_2_ was selected to mimic instances where tissues grow very rapidly, such as white adipose tissue during progression of obesity, and this tension has been used for previous *in vitro* studies (Lempesis et al., 2020; Emami et al., 2021).

**Fig. 1:**
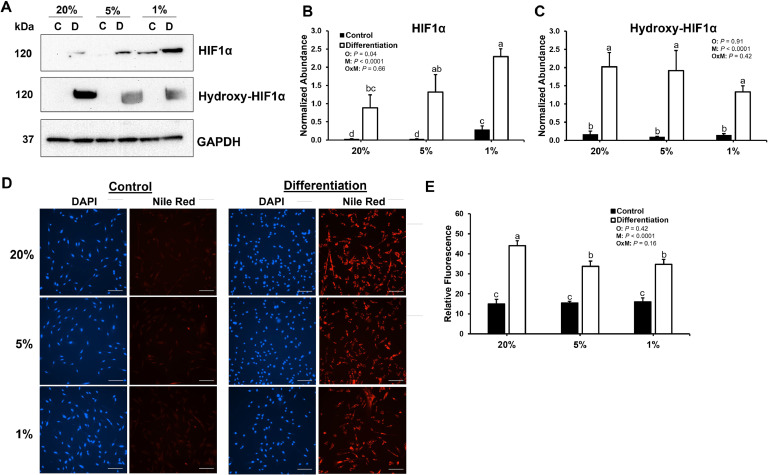
Effects of different media and oxygen tensions on lipid accumulation in broiler preadipocytes. **(A)** Representative images of western blots for HIF1α and hydroxy-HIF1α for control (C) and differentiation (D) cultures at 20%, 5%, or 1% O_2_. GAPDH was used as the housekeeping protein. Densitometry of **(B)** HIF1α and **(C)** hydroxy-HIF1α was performed for three independent replicates. Different lowercase letters above the bars represent statistical differences between groups (*P* < 0.05). *P* values are provided for factors and interactions. **(D)** Representative images of Nile Red staining to detect lipid for control and differentiation cultures at 20%, 5%, or 1% O_2_ and **(E)** quantification of relative fluorescence for at least five images per replicate (n=3). Scale bars represent 100 μm. Different lowercase letters above the bars represent statistical differences between groups (*P* < 0.05). *P* values are provided for factors and interactions. O, oxygen tension; M, medium; OxM, interaction between O and M.

First, abundance of HIF1α and hydroxy-HIF1α proteins, the hydroxylated form tagged for degradation, were measured in the preadipocytes cultured at 20%, 5%, or 1% O_2_ in control or differentiation medium to validate the hypoxia treatment (Fig. 1A, B, and C). For control cultures, preadipocytes cultured at 1% O_2_ had increased abundance of HIF1α protein compared to cultures at 20% or 5% O_2_ (*P* < 0.05). For differentiation cultures, preadipocytes cultured at 1% O_2_ had increased levels of HIF1α protein compared to cultures at 20% O_2_ (*P* < 0.05), confirming that a hypoxic state was achieved. Differentiation cultures had increased abundance of HIF1α and hydroxy-HIF1α protein compared to control cultures except for HIF1α abundance between differentiation at 20% O_2_ and control at 1% O_2_. Although it was not measured, these increases are likely due to the presence of insulin in the differentiation medium, which has been shown to enhance abundance of HIFα in 3T3-L1 adipocytes (He et al., 2011).

To quantify intracellular lipid, preadipocyte cultures were stained with Nile Red, which is highly lipophilic. As expected, increased accumulation of lipid was observed in broiler preadipocytes cultured in differentiation medium compared to the control medium (*P* < 0.05; Fig. 1D and E). Interestingly, lipid accumulation was increased in preadipocytes that were cultured in differentiation medium at atmospheric (20%) O_2_ compared to physiological (5%) or hypoxic (1%) O_2_ tensions (*P* < 0.05), thus demonstrating an inverse relationship with HIF1α abundance. Although conflicting reports exist on the effect of oxygen tension on adipogenesis, Lempesis et al. (2020) reported that severe hypoxia (∼1% O_2_) for 24 hours or less decreased fatty acid uptake and subsequent lipogenesis in adipocytes. Additionally, obese human subjects were observed to have increased oxygen tensions in adipose tissue as compared to their lean counterparts (Goossens et al., 2011). The increased oxygen tension in adipose tissue of obese individuals has been linked to mitochondrial dysfunction, which reduces oxygen consumption by the cell. Along these lines, adipogenic differentiation of chicken preadipocytes may be promoted in hyperoxic conditions *in vitro*. Although broilers are animals that have high metabolic activity and prone to obesity (Wang et al., 2017), the exact impact of oxygen tension on adipogenesis *in vivo* is still unknown.

**Fig. 2:**
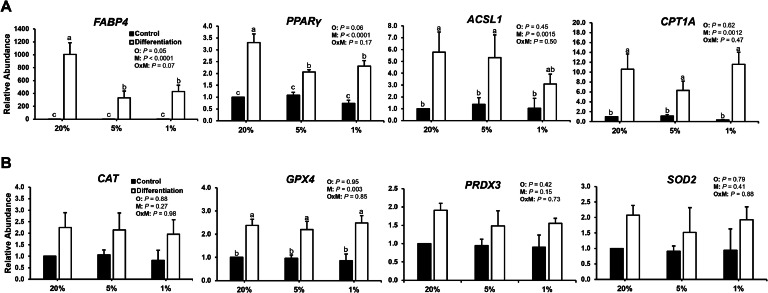
Relative abundance of transcripts related to **(A)** fatty acid transport (*FABP4*), adipogenesis (*PPARγ*), and lipid metabolism (*ASCL1* and *CPT1A*) as well as **(B)** antioxidant function (*CAT, GPX4, PRDX3*, and *SOD2*) between control and differentiation groups at 20%, 5%, or 1% O_2_. β-actin was used as a housekeeping gene, and four replicates were assessed. Different lowercase letters above the bars represent statistical differences between groups (*P* < 0.05). *P* values are provided for factors and interactions. O, oxygen tension; M, medium; OxM, interaction between O and M.

Subsequently, abundance of transcripts related to fatty acid transport and metabolism was analyzed in the preadipocyte cultures to determine the effect of oxygen tension on these processes during differentiation *in vitro*. In addition to the increased lipid accumulation in differentiated preadipocytes cultured at 20% O_2_, abundances of fatty acid-binding protein 4 (*FABP4*) and peroxisome proliferator-activated receptor gamma (*PPARγ*) mRNA were also increased in cultures at 20% O_2_ compared to 5% or 1% O_2_, and all differentiation cultures had increased abundance compared to control cultures (*P* < 0.05; Fig. 2A). Fatty acid-binding protein 4 is involved in the intracellular transport of fatty acids, facilitating their storage and utilization, and PPARγ is a key regulator of adipocyte differentiation and lipid storage. Furthermore, expression patterns of FABP4 and PPARγ have been shown to increase during development of adipose tissue in chickens (Sato et al., 2009; Chen et al., 2014). Abundance of *ACSL1*, which encodes an enzyme to convert fatty acids into acyl-CoAs for intracellular lipid synthesis or beta oxidation, was increased in differentiated preadipocytes cultured at 20% or 5% O_2_ (*P* < 0.05) but not at 1% O_2_ compared to the controls, indicating potential inhibition by hypoxia. However, abundance of *CPT1A*, which encodes the key enzyme for mitochondrial uptake and beta-oxidation of lipids, was increased in all differentiated groups compared to the controls (*P* < 0.05); thus, oxygen tension appears to regulate lipid transport and adipogenesis but not lipid metabolism in broiler preadipocytes *in vitro*.

Differences in lipid accumulation and abundance of adipogenesis-related transcripts were not observed between differentiation cultures at 5% or 1% O_2_ which may have occurred for several reasons. First, tissues have different oxygen tensions, and some tissues, such as adipose, can have a wide range (Lempesis et al., 2020). Therefore, incubating preadipocytes at 5% O_2_ versus 1% O_2_ may not have led to a substantial difference in intracellular oxygen tension. This is further supported by the fact that 5% O_2_ can be considered mild hypoxia, and no differences in HIF1α protein abundance were observed between the differentiation cultures at 5% O_2_ or 1% O_2_. However, differences were observed in HIF1α protein abundance between cultures at 20% O_2_ and 1% O_2_ but not 20% O_2_ and 5% O_2_. Second, as mentioned previously, culture in 20% O_2_ represents supraphysiological levels, and hyperoxia may have resulted in cellular changes in lipid synthesis and transport that would not normally occur in the body. In human primary preadipocytes, culture at atmospheric (20%) O_2_ increased lipid droplet size compared to 10% O_2_ or 5% O_2_, and cultures at 10% O_2_ and 5% O_2_ had increased abundance of markers for lipolysis (Famulla et al., 2012), which were not measured in the present study. Lastly, the step-down approach may have allowed for adaptation to hypoxia in the 1% O_2_ cultures; therefore, a more rapid step-down could be tested in these cultures, but induction of necrosis needs to be monitored.

Abundance of antioxidant-related transcripts was assessed to determine the effect of adipogenic differentiation and varying oxygen tensions on redox status and intracellular stress. Specifically, culture at atmospheric (20%) O_2_ tension may lead to a hyperoxic intracellular state and increased production of ROS. Synthesis of antioxidant enzymes is needed to control ROS levels and prevent intracellular damage. Abundance of glutathione peroxidase 4 (*GPX4*) was increased in all the differentiated cultures compared to the control cultures, regardless of oxygen tension; however, differences in the other transcripts encoding antioxidant enzymes (*CAT, PRDX3, SOD2*) were not observed (Fig. 2B). Increased metabolism through beta-oxidation in the differentiation groups may have resulted in the upregulation of *GPX4* as a protective response to regulate the intracellular concentration of hydrogen peroxide (H_2_O_2_), which can be reduced to hydroxyl radicals that contribute to membrane and DNA damage (Trujillo et al., 2023). Moreover, GPX4 is critical in mitigating lipid peroxidation (Trujillo et al., 2023), serving to protect against this process during increased lipid accumulation and metabolism in the differentiation cultures.

In the current study, preadipocyte cultures from commercially relevant broiler embryos were established to understand the role of oxygen tension during adipogenic differentiation. Typically, chicken preadipocyte cultures are performed in incubators that use atmospheric oxygen tension (∼20-21%), which may not fully recapitulate the physiological state as adipose oxygen tension has been shown to be approximately 3-11% (Lempesis et al., 2020). Moreover, most studies examining the impact of oxygen tension on adipogenesis have been conducted in humans or other mammals; therefore, little is known regarding this process in avian species. Culture of broiler preadipocytes at atmospheric oxygen tension in this study seemed to alter, through interconnected mechanisms, cellular and metabolic signaling, thereby promoting increased lipid accumulation. Additionally, accumulation of lipid in preadipocytes cultured in differentiation medium upregulated *GPX4*, which is involved in the cellular protective mechanism against lipid peroxidation. In summary, aside from medium composition, other factors, including oxygen tension, can directly impact differentiation of broiler preadipocytes *in vitro*.

## Declaration of competing interest

The authors declare that no conflict of interest exists.
